# Efficacy analysis of disitamab vedotin (RC-48) in the treatment of HER2-low metastatic breast cancer: a case report

**DOI:** 10.3389/fonc.2026.1652716

**Published:** 2026-05-04

**Authors:** Ruiyue Wu, Xi Chen, Tong Li, Yunmei Zhang, Xiaoling Ling

**Affiliations:** 1 The First Clinical Medical College of Lanzhou University, Lanzhou, Gansu, China; 2 Breast Tumor Center, Guangdong Provincial Key Laboratory of Malignant Tumor Epigenetics and Gene Regulation, Medical Research Center, Sun Yat-sen Memorial Hospital, Sun Yat-sen University, Guangzhou, Guangdong, China; 3 Department of Oncology, The First Hospital of Lanzhou University, Lanzhou, Gansu, China

**Keywords:** antibody-drug conjugates (ADCs), breast cancer, case report, HER2-low, targeted therapy

## Abstract

HER2-low breast cancer, defined as immunohistochemistry (IHC) 1+ or 2+ with negative *in situ* hybridization (ISH), has emerged as a clinically relevant therapeutic subgroup. We report the case of a 40-year-old woman with heavily pretreated HER2-low metastatic breast cancer who received disitamab vedotin (RC-48) after failure of multiple prior systemic therapies. During the disease course, HER2 status changed dynamically from HER2-low at initial diagnosis to HER2–0 after metastasis, and then to HER2-low again after further progression, highlighting the biological heterogeneity of metastatic breast cancer and the importance of repeat biopsy. Following RC-48 treatment, chest-wall cutaneous lesions regressed markedly, tumor markers showed partial biochemical improvement, and imaging demonstrated substantial shrinkage of visceral metastatic lesions. The time to treatment failure (TTF) was 4.6 months. After treatment discontinuation following a COVID-19-associated septic shock event, the patient declined further anticancer therapy and subsequently died 13.7 months after RC-48 initiation. Therefore, survival outcomes should be interpreted cautiously. As the first China-developed anti-HER2 antibody-drug conjugate (ADC), RC-48 may provide clinically meaningful benefit in selected patients with HER2-low metastatic breast cancer. This case also underscores the importance of reassessing HER2 status during disease evolution and supports the potential value of ADC-based therapy in later-line treatment.

## 1 Introduction

Breast cancer is the most common malignancy worldwide, and intratumoral heterogeneity is a critical factor influencing its progression and prognosis (1, 2). Human Epidermal Growth Factor Receptor 2 (HER2)-low expression is defined as an immunohistochemistry (IHC) score of 1+ or 2+ with negative *in situ* hybridization (ISH) results (3). Historically, such cases were classified as HER2-negative and thus ineligible for HER2-targeted therapies (4). Following the groundbreaking results of the DESTINY-Breast04 trial demonstrating the efficacy of trastuzumab deruxtecan (T-DXd) in HER2-low breast cancer (5), T-DXd has been incorporated into treatment recommendations for HER2-low triple-negative breast cancer as a second-line therapy in both the Chinese Society of Clinical Oncology (CSCO) Breast Cancer Guidelines (2025 edition) and the National Comprehensive Cancer Network (NCCN) Guidelines (Version 4, 2025) (6). Disitamab vedotin (RC-48), the first China-developed anti-HER2 ADC, is currently under clinical investigation for HER2-low breast cancer (7). This study presents a case of a 40-year-old female diagnosed with HER2-low breast cancer during treatment, who achieved significant clinical response following anti-HER2 ADC therapy.

## 2 Case presentation

In May 2014, a 40-year-old woman underwent surgery for a right breast mass, which was pathologically confirmed as invasive ductal carcinoma. Immunohistochemistry demonstrated estrogen receptor (ER) positivity, progesterone receptor (PR) positivity, and HER2 IHC 1 +. No distant metastases were detected on baseline imaging. The patient underwent right breast lumpectomy with axillary lymph node dissection (ypT2N2M0), followed by six cycles of adjuvant chemotherapy (pirarubicin, docetaxel, and cyclophosphamide), radiotherapy to the right chest wall and supraclavicular region, and subsequent endocrine therapy. She achieved a disease-free survival (DFS) of 64 months.

In September 2019, radiologic progression was identified with axillary lymph node, chest wall, and bone metastases. Biopsy of metastatic lesions revealed ER-negative, PR-negative, and HER2 IHC 0 status. Re-biopsy of metastatic lesions was performed at each documented disease progression to reassess ER, PR, and HER2 expression. Accordingly, receptor reassessment was conducted in September 2019 at the time of initial metastatic progression and again in November 2020 due to further disease progression and treatment resistance. The rationale for repeat biopsy was to evaluate potential receptor conversion that might influence subsequent therapeutic strategies, particularly eligibility for HER2-targeted therapy.

**Figure 1:**
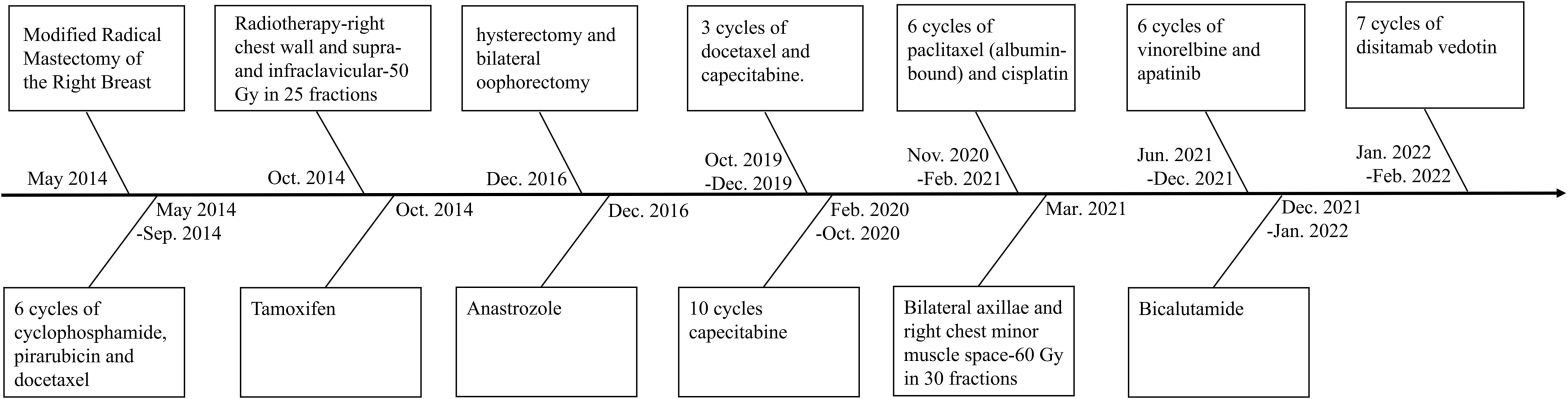
Complete treatment regimen of the patient.

According to the Chinese Society of Clinical Oncology (CSCO) Breast Cancer Guidelines (8), the patient received three cycles of docetaxel plus capecitabine, followed by ten cycles of capecitabine and radiotherapy. By November 2020, imaging demonstrated progression with increased bilateral axillary lymphadenopathy. Repeat biopsy confirmed ER-negative, PR-negative, and HER2 IHC 2+ with negative *in situ* hybridization (ISH), consistent with HER2-low status. In accordance with CSCO guidelines (8), six cycles of albumin-bound paclitaxel combined with cisplatin were administered alongside radiotherapy. In June 2021, a new right chest wall nodule was detected. After multidisciplinary team (MDT) discussion, the patient was treated with six cycles of vinorelbine plus apatinib. The complete treatment timeline is illustrated in **Figure 1** and **Supplementary Table 1**.

**Figure 2:**
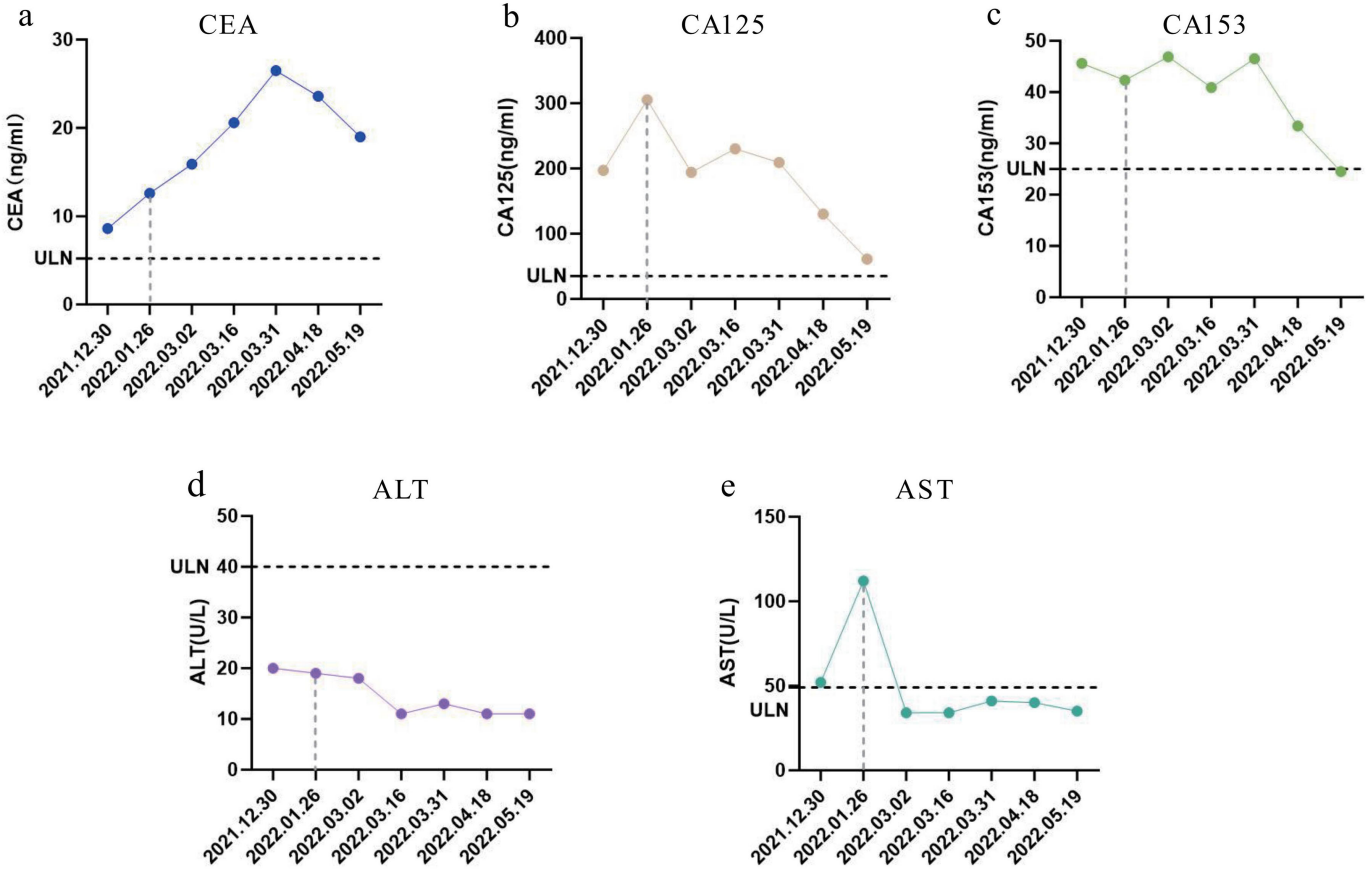
Changes in tumor markers and liver enzymes. **(A)** Carcinoembryonic antigen (CEA); **(B)** Cancer antigen 125 (CA125); **(C)** Carbohydrate antigen 15-3 (CA153); **(D)** Alanine aminotransferase (ALT); **(E)** Aspartate aminotransferase (AST). ULN, Upper limit of normal.

**Figure 3:**
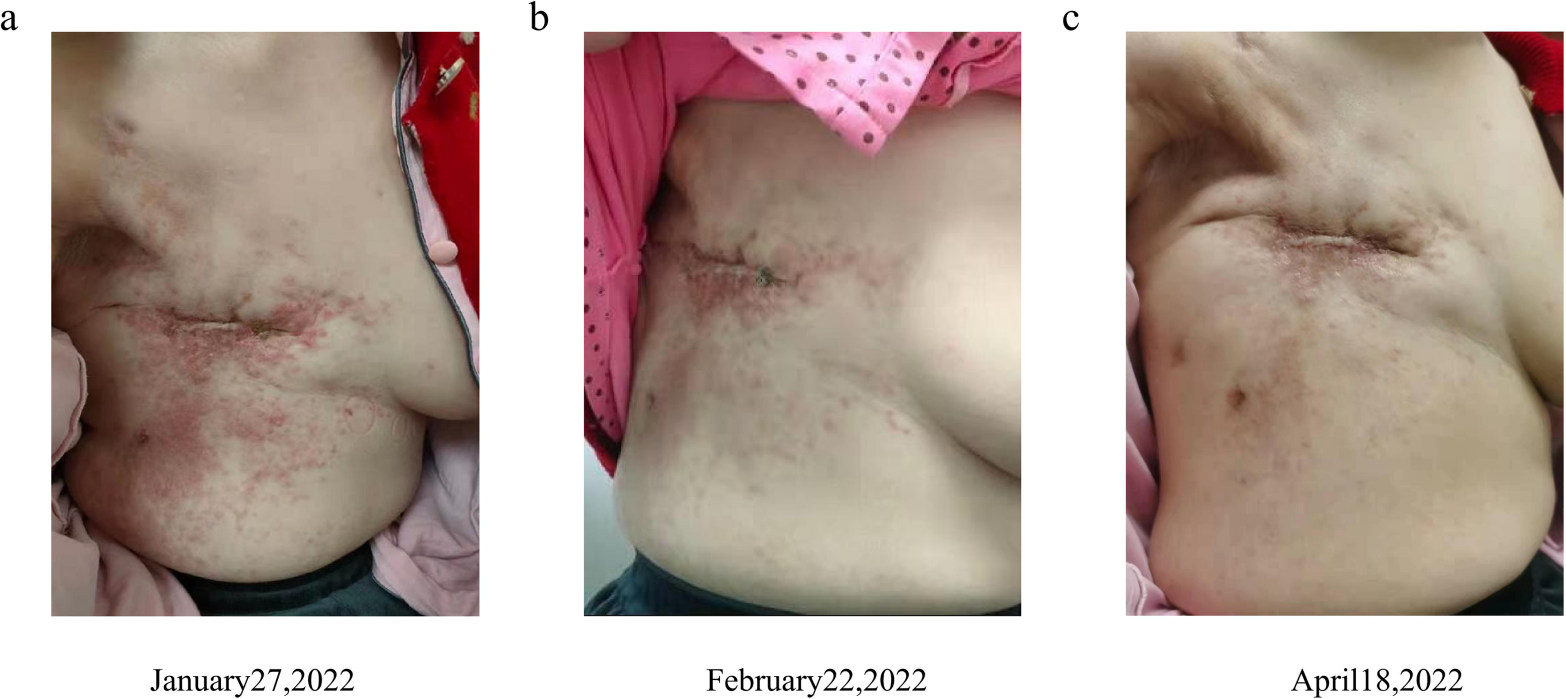
Cutaneous infiltration of the chest wall. **(A)** Chest wall cutaneous infiltration before the first administration of RC-48 on January 27, 2022; **(B)** chest wall cutaneous infiltration after two cycles of RC-48 on February 22, 2022; **(C)** chest wall cutaneous infiltration after five cycles of RC-48 on April 18, 2022.

**Figure 4:**
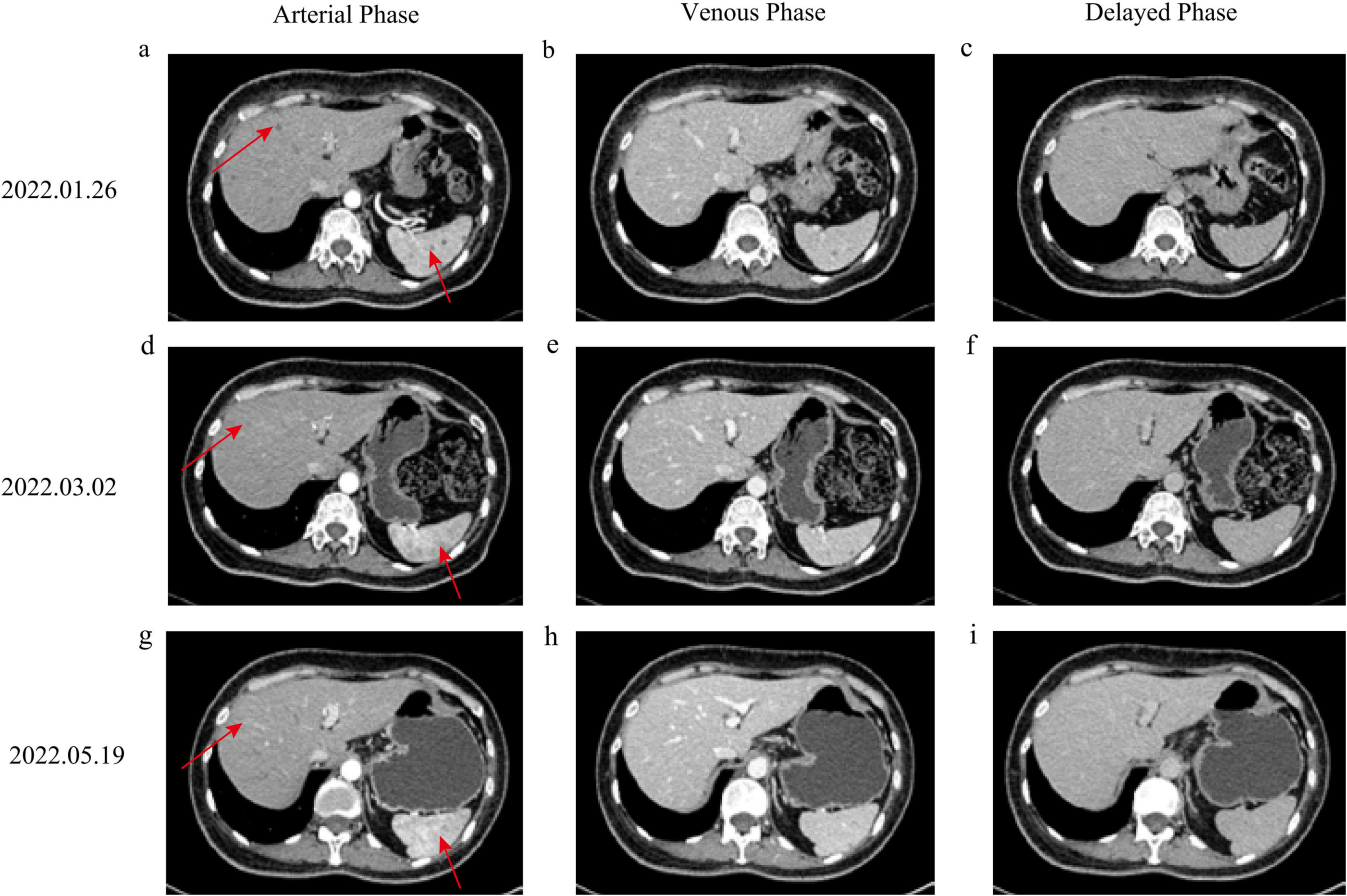
Imaging changes. **(A–C)** Pre-RC-48 images obtained on January 26, 2022; **(D–F)** images after two cycles of RC-48 on March 2, 2022; **(G–I)** images after six cycles of RC-48.

In December 2021, disease progression was evidenced by enlargement of right chest wall nodules and rising tumor marker levels (CA153: 45.6 ng/mL; CEA: 12.6 ng/mL; CA125: 197 ng/mL). Biopsy confirmed androgen receptor (AR) positivity, and bicalutamide therapy was initiated (9). By January 2022, further progression was observed, with additional elevation of tumor markers (CA153: 46.9 ng/mL; CEA: 15.9 ng/mL; CA125: 305 ng/mL; **Figures 2B, C**). Multiple miliary cutaneous nodules developed on the ipsilateral chest wall (**Figure 3A**) and were confirmed as metastatic lesions by histopathology and immunohistochemistry. Contrast-enhanced computed tomography (CT) scans demonstrated newly developed liver and splenic metastases (**Figures 4A–C**), accompanied by elevated aspartate aminotransferase (AST, 112 U/L; **Figures 2D, E**).

Following multidisciplinary team (MDT) evaluation, RC-48 was initiated on January 28, 2022. The drug was administered intravenously at a dose of 2.0 mg/kg every two weeks (q2w), in accordance with the recommended dosing schedule. Each cycle consisted of a single 90-minute infusion. A total of six cycles were delivered between January 28 and June 17, 2022, without dose reduction or treatment delay prior to the COVID-19-associated event. Treatment response was assessed using contrast-enhanced CT of the chest and abdomen, along with liver magnetic resonance imaging (MRI) when indicated. Baseline imaging was performed prior to RC-48 initiation, and follow-up imaging was conducted after every two cycles. The last documented CT scan was performed on May 19, 2022.

Chest-wall nodules regressed (**Figures 4D–F**), tumor markers showed overall improvement (CA153: 33.4 ng/mL; CEA: 23.6 ng/mL; CA125: 130 ng/mL; **Figures 2A–C**), and AST declined from 112 U/L at baseline to 59 U/L during treatment (**Figures 2D, E**). Given that AST elevation was already present before RC-48 initiation in the setting of newly developed liver metastases, and improved in parallel with radiologic regression of hepatic lesions, this change was interpreted as being more consistent with improvement of metastatic liver involvement than with treatment-related hepatotoxicity.

During RC-48 therapy, the patient developed mild temporomandibular joint discomfort after the infusion on March 16, 2022. The symptom did not impair mouth opening or oral intake and was managed conservatively with local acupuncture and massage, resulting in partial relief. In addition, grade 1 neutropenia was observed according to CTCAE version 5.0 criteria. The event resolved within one week following short-term granulocyte colony-stimulating factor support, without treatment interruption or dose modification. No other clinically significant adverse events were documented.

After completion of the sixth cycle of RC-48 on June 17, 2022, the patient developed COVID-19–associated septic shock (presenting with fever and altered consciousness) and received intensive care including antiviral therapy and organ support. Prior to this event, the patient had achieved a significant clinical response to RC-48. The last documented CT scan was performed on May 19, 2022 (**Figures 4G–I**), which revealed no evidence of liver or splenic metastatic lesions. Concurrently, tumor markers showed substantial improvement (CA153: 24.5 ng/mL; CA125: 61.1 ng/mL; CEA: 19 ng/mL), and liver function remained within normal limits (**Figures 2D, E**).

## 3 Results and follow-up

The COVID-19-associated septic shock was considered unrelated to RC-48 therapy. After recovery from this critical illness, the patient declined further anticancer treatment and subsequently received supportive care only. The time to treatment failure (TTF), defined as the interval from RC-48 initiation on January 28, 2022 to the last administration on June 17, 2022, was 4.6 months. The last documented CT evaluation was performed on May 19, 2022 and demonstrated ongoing radiologic response. The patient died on March 22, 2023, corresponding to 13.7 months from RC-48 initiation to death. However, because no further anticancer therapy was administered after treatment discontinuation and death was not directly attributable to documented tumor progression during RC-48 therapy, this survival duration should be interpreted cautiously and may not accurately reflect the treatment-related survival benefit of RC-48.

## 4 Discussion

This case demonstrates the meaningful clinical benefit of RC-48 in a patient with HER2-low metastatic breast cancer who had previously received five lines of systemic therapy. These included anthracycline- and taxane-based chemotherapy, platinum-based chemotherapy, endocrine therapy, anti-androgen therapy, and anti-angiogenic treatment, reflecting a heavily pretreated disease setting with limited remaining therapeutic options.

At the time of treatment initiation in January 2022, trastuzumab deruxtecan (T-DXd) had not yet become widely accessible in our region. RC-48 was available and supported by emerging clinical data in HER2-positive and HER2-low breast cancer. Considering the patient’s rapidly progressive liver metastases, limited remaining systemic options, and confirmed HER2-low status, the multidisciplinary team (MDT) determined that RC-48 represented the most feasible and evidence-supported antibody-drug conjugate choice in this clinical context.

Throughout the disease course, the patient exhibited dynamic changes in receptor status, shifting from HR-positive/HER2-low (IHC 1+) at initial diagnosis to triple-negative/HER2-0 (IHC 0) at recurrence, and later to HR-negative/HER2-low (IHC 2+) after further progression. This pattern likely reflects not only temporal and spatial intratumoral heterogeneity, but also treatment-driven clonal selection during disease evolution (10, 11). Increasing evidence suggests that HER2-low expression may represent a dynamic phenotypic state rather than a fixed biological subtype, with HER2-positive, HER2-low, and HER2–0 subclones potentially coexisting within the same tumor and being differentially enriched under therapeutic pressure. In this context, the loss and subsequent re-emergence of HER2-low expression in our patient may be understood as a manifestation of tumor clonal evolution rather than a simple laboratory fluctuation.

Clinically, this dynamic conversion had direct therapeutic implications. The re-identification of HER2-low expression at a later stage reopened the possibility of HER2-targeted ADC therapy and ultimately enabled treatment with RC-48, from which the patient derived substantial benefit. This observation further supports the value of repeat biopsy in metastatic breast cancer, particularly in heavily pretreated patients, because reassessment of HER2 status may capture evolving targetable subclones and expand treatment opportunities in the ADC era.

Following initiation of RC-48, the patient achieved a rapid and substantial response across multiple metastatic sites, including the liver, spleen, lymph nodes, and extensive chest-wall cutaneous lesions, all of which are disease patterns typically associated with chemoresistance and poor prognosis. The concurrent decline in AST, which had been elevated at baseline in association with newly developed liver metastases, further supported the interpretation that liver function improvement was secondary to regression of hepatic tumor burden rather than an apparent signal of drug-induced liver injury. Although radiologic improvement of lymph node disease was observed, the site-specific response of lymph node metastases was not systematically quantified in this case; therefore, these findings should be interpreted cautiously and do not allow a definitive site-specific conclusion regarding lymph node metastases. The marked clinical improvement aligns with the pharmacological properties of disitamab vedotin, which combines a high-affinity anti-HER2 antibody with a cleavable linker and MMAE payload capable of exerting a potent bystander effect (12, 13). These real-world findings are consistent with results from prior clinical trials demonstrating that RC-48 is effective even in patients who have undergone multiple lines of therapy, and support the concept that low-level HER2 expression is sufficient to mediate meaningful ADC uptake and antitumor activity in HER2-low breast cancer (14, 15). The mechanism underlying the activity of RC-48 in HER2-low tumors is not yet fully understood. A plausible explanation is that disitamab vedotin retains sufficient binding and internalization even in the setting of low HER2 expression, thereby enabling intracellular release of the MMAE payload. In addition, the membrane-permeable nature of MMAE may produce a bystander effect, allowing cytotoxic activity to extend to adjacent tumor cells with minimal or heterogeneous HER2 expression. This may be particularly relevant in metastatic breast cancer, where spatial heterogeneity and dynamic HER2 modulation are common. However, the precise molecular determinants of RC-48 sensitivity in HER2-low disease remain to be further investigated.

Although CA125 is not routinely used for monitoring breast cancer, elevated levels have been reported in advanced metastatic disease, particularly with extensive visceral involvement. In this case, CA125 elevation was interpreted as a non-specific marker of tumor burden rather than a feature of HER2-low biology. Its parallel decline with other tumor markers and radiologic improvement further supported its correlation with disease activity.

This case provides several important clinical insights. First, reassessment of HER2 status at progression should be considered standard practice in metastatic breast cancer, particularly for HER2-low disease, where even small changes in expression can open the door to ADC therapy. In this patient, the re-emergence of HER2-low expression directly enabled treatment with RC-48 and contributed to significant disease control. Second, the rapid regression of chest-wall skin metastases-typically refractory to systemic chemotherapy-highlights the potential of ADCs, via their bystander effect, to address locally aggressive or skin-dominant patterns of metastasis. Third, the patient’s AR-positive, chemotherapy-refractory phenotype highlights the molecular complexity of advanced breast cancer and the need for alternative therapeutic strategies in biologically heterogeneous subtypes (16).

Nevertheless, several limitations warrant consideration. As a single-patient case report, the findings cannot be generalized, and the observed therapeutic response may be influenced by individual tumor biology. Additionally, the lack of serial comprehensive molecular profiling, such as next-generation sequencing or transcriptomic assessment, limited our ability to directly characterize the clonal evolution underlying HER2 status shifts and to further explore the molecular basis of ADC responsiveness. Finally, because RC-48 was discontinued after a COVID-19-associated septic shock event and the patient subsequently declined further anticancer treatment, survival outcomes in this case should be interpreted cautiously. In this context, the time to treatment failure (TTF, 4.6 months) may better reflect the actual duration of RC-48 exposure, whereas the interval from RC-48 initiation to death (13.7 months) may not accurately represent the treatment-specific survival benefit. The absence of further anticancer therapy and the lack of subsequent radiologic assessment also prevented evaluation of the maximal duration of treatment benefit and long-term safety. These limitations highlight the need for prospective studies to further define the optimal timing, duration, and molecular predictors of response to ADC therapy in HER2-low MBC.

In summary, this case supports the real-world activity of RC-48 in heavily pretreated HER2-low metastatic breast cancer and underscores the importance of reassessing HER2 status during disease evolution. The observed regression of visceral and chest-wall cutaneous lesions suggests that next-generation ADCs may offer meaningful benefit in biologically complex metastatic disease. However, the activity of RC-48 in specific metastatic sites, including lymph node-dominant disease, requires further evaluation in larger studies.

## Data Availability

The original contributions presented in the study are included in the article/**Supplementary Material**. Further inquiries can be directed to the corresponding author.
