# Supplementary material for: Efficacy analysis of disitamab vedotin (RC-48) in the treatment of HER2-low metastatic breast cancer: a case report
Source: Front Oncol. 2026 May 4;16:1652716. doi: 10.3389/fonc.2026.1652716 (PMC13180562; doi:10.3389/fonc.2026.1652716)
Supplement: Supplementary file 1 [file DataSheet1.docx]

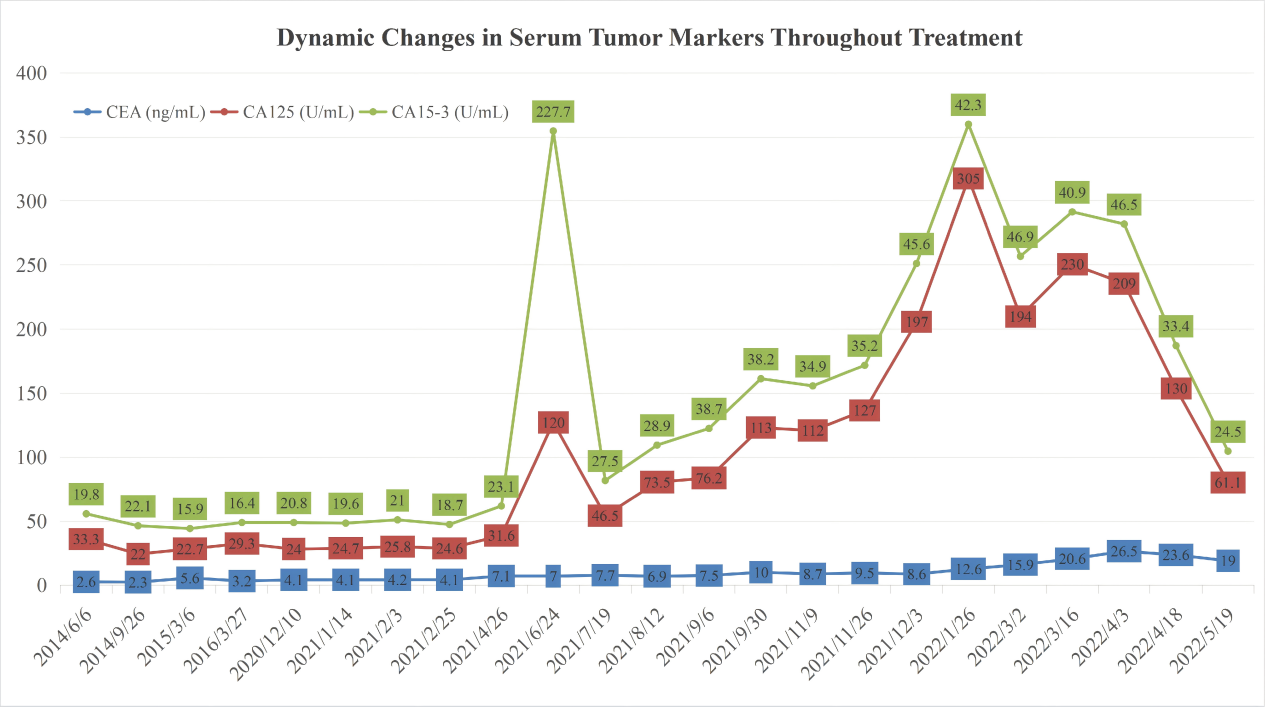


Supplementary Figure1. Dynamic Changes in Serum Tumor Markers Throughout Treatment Serial serum levels of CEA, CA125, and CA15-3 during the available clinical course are shown. The upper limits of normal (ULN) were: 2014–2016, CEA <4.7 ng/mL, CA125 <35.0 U/mL, and CA15-3 <25.0 U/mL; 2020 onward, CEA <5.2 ng/mL, CA125 <35.0 U/mL, and CA15-3 <25.0 U/mL.
