# Supplementary material for: Efficacy analysis of disitamab vedotin (RC-48) in the treatment of HER2-low metastatic breast cancer: a case report
Source: Front Oncol. 2026 May 4;16:1652716. doi: 10.3389/fonc.2026.1652716 (PMC13180562; doi:10.3389/fonc.2026.1652716)
Supplement: Supplementary file 2 [file Table1.docx]

**Supplementary Table 1. Summary of the Patient’s Treatment History and Clinical Outcomes**

| Period | Line of Therapy | Disease Status | Treatment Regimen | Dose & Schedule | Best Response (RECIST 1.1) | | Adverse Events (CTCAE v5.0) | Outcome |
| --- | --- | --- | --- | --- | --- | --- | --- | --- |
| May 2014 | — | Primary breast cancer (ER+, PR+, HER2 1+) | Lumpectomy + ALND | — | IDC, ypT2N2M0 | — | | Completed |
| Jun–Sep 2014 | Adjuvant | Postoperative | Anthracycline + Taxane + Cyclophosphamide | q3w ×6 | NED | Grade 1 GI toxicity | | Completed |
| Oct 2014 | Adjuvant | Postoperative | Radiotherapy | 50 Gy/25F | — | Mild GI toxicity | | Completed |
| 2014–2019 | Endocrine | HR+ | Aromatase inhibitor | Daily | DFS 64 months | Well tolerated | | — |
| Sept 2019–Jan 2020 | 1st-line metastatic | LN + bone metastases (HER2 0) | Docetaxel + Capecitabine | q3w | SD | Grade 3 neutropenia | | PD |
| Feb 2020–Nov 2020 | 2nd-line | Metastatic | Capecitabine | q3w | SD | Grade 1 GI toxicity | | PD |
| Nov 2020–Jun 2021 | 3rd-line | HER2 2+/ISH- (HER2-low) | Nab-paclitaxel + Cisplatin | q3–4w | SD | Grade 1–2 hematologic | | PD |
| Jun 2021–Dec 2021 | 4th-line | Chest wall metastasis | Vinorelbine + Apatinib | Standard | SD | Manageable | | PD |
| Dec 2021–Jan 2022 | 5th-line | AR+ | Bicalutamide | Oral daily | PD | Well tolerated | | Switched |
| Jan 28–Jun 17 2022 | 6th-line (RC-48) | Liver + splenic metastases (HER2-low) | Disitamab vedotin | **2.0 mg/kg IV q2w ×6 cycles** | **Partial response (PR)** | Grade 1 neutropenia; mild TMJ discomfort | | Treatment stopped due to COVID-19 |
| Jun 2022 | — | COVID-19 septic shock | ICU support | — | — | — | | Death Mar 2023 |
